# Efficacy of early administration of thrombomodulin alfa in patients with sepsis-induced disseminated intravascular coagulation: subanalysis from post-marketing surveillance data

**DOI:** 10.1186/cc13438

**Published:** 2014-03-17

**Authors:** Y Eguchi, S Gando, H Ishikura, D Saitoh, J Mimuro, H Takahashi, I Kitajima, H Tsuji, T Matsushita, Y Sakata

**Affiliations:** 1 Shiga University of Medical Science, Shiga, Japan; 2 Hokkaido University Graduate School of Medicine, Hokkaido, Japan; 3 Fukuoka University, Fukuoka, Japan; 4 National Defense Medical College, Saitama, Japan; 5 Jichi Medical University, School of Medicine, Tochigi, Japan; 6 Niigata Prefectural Kamo Hospital, Niigata, Japan; 7 Graduate School of Medical and Pharmaceutical Science, University of Toyama, Japan; 8 Kyoto Prefectural University of Medicine, Kyoto, Japan; 9 Nagoya University Hospital, Nagoya, Japa

## Introduction

We hypothesize that the early administration of thrombomodulin alfa (TM-alfa) (Recomodulin^® ^injection; Asahi Kasei Pharma Corporation, Tokyo, Japan) could improve mortality in patients with sepsis-induced disseminated intravascular coagulation (DIC). TM-alfa has been approved for use as a curative medicine for DIC in Japan from 2008 that was examined in a multicenter, randomized, clinical trial [1].

## Methods

DIC was diagnosed based on the Japanese Association for Acute Medicine (JAAM) criteria. From May 2008 to April 2010, a total of 1,787 patients with sepsis-induced DIC from post-marketing surveillance data [2] were analyzed. The survival rates on day 28 after the last TM-alfa administration were evaluated.

## Results

The 28-day survival rate was 64.5%. Use of other anticoagulants before and after TM-alfa administration did not affect the survival rate (present vs. absent: 63.8% vs. 65.2% (*P *= 0.782) and 62.5% vs. 65.3% (*P *= 0.606) respectively). The survival rate decreased significantly in proportion to the duration of DIC before TM-alfa administration (*P *< 0.001). More precisely, the 28-day survival rate was 66.4% when TM- alfa was injected on the same day that DIC was diagnosed, whereas it was 48.5% and 31.1% when TM-alfa was started 4 days later and 7 days or more after DIC was diagnosed, respectively. These differences were significant (*P *= 0.033 and *P *< 0.001, respectively).

## Conclusion

The early administration of TM-alfa may be effective for patients with sepsis-induced DIC diagnosed based on the JAAM criteria.

## References

[B1] Saito H J Thromb Haemost 2007 5 31 41 10.1111/j.1538-7836.2006.02267.x 17059423

[B2] Mimuro J Thromb Res 2013 131 436 443 10.1016/j.thromres.2013.03.008 23566534

